# Optimization of cassava (*Manihot esculenta* Crantz) grafting technique to enhance its adoption in cassava cultivation

**DOI:** 10.1016/j.mex.2024.102904

**Published:** 2024-08-19

**Authors:** Frank Opoku-Agyemang, Jacqueline Naalamle Amissah, Stella Owusu-Nketia, Peter Amoako Ofori, Michitaka Notaguchi

**Affiliations:** a College of Basic and Applied Sciences (CBAS), University of Ghana (UG), P.O. Box LG 25, Legon, Accra, Ghana; b Graduate School of Bioagricultural Sciences, Nagoya University, Furo-Cho, Chikusa-Ku, Nagoya 464-8601, Japan; c Department of Botany, Graduate School of Science, Kyoto University, Kitashirakawa Oiwake-cho, Sakyo-ku, Kyoto 606-8502, Japan

**Keywords:** Grafting, Cassava, Cleft grafting, Wooden-healing chamber, Rootstock, Scion, Cassava grafting using top wedge technique and healing grafts in a wooden chamber

## Abstract

Grafting techniques have been successfully adopted to improve resistance to biotic and abiotic stresses, increase yields, fruit quality and study systemic signaling in plants. This technique has not been fully explored in cassava and there is currently no standardized grafting method for this species published especially in Africa. This is the first report on cassava grafting protocol in Africa with valuable advantages including utilizing a cost-effective and environmentally friendly wooden healing chamber. In this study, we describe an optimized cleft grafting protocol for cassava utilizing a wooden healing chamber and outline the step-by-step procedure with optimum conditions to generate a high grafting success rate. Using a top wedge grafting technique with high reproducibility and success rates, we developed a straightforward and robust grafting protocol for cassava (*M. esculenta*) cultivars. Grafting success was recorded and this protocol produced a high grafting success of 90 % and its reproducibility makes it suitable for mass production thereby addressing the need for efficient cassava propagation. This grafting protocol requires less specialized equipment and expertise making it more accessible to farmers and researchers with limited resources to promote the use of grafting for cassava growth, yield improvement and advanced studies such as systemic long-distance signaling in plants.

• Optimization of cleft grafting method obtains a high success grafting rate of cassava.
• A wooden healing chamber provides a controlled environment for graft healing.
• Promoting cassava grafting; a priority to produce new cultivars and explore breeding research prospects.

## Graphical abstract

**Figure:**
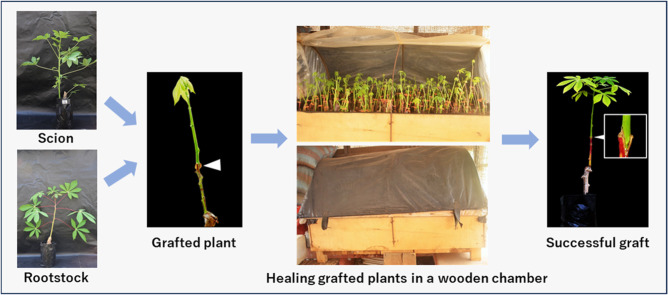

Specifications table

**Table:** 

Subject area:	*Agricultural and Biological Sciences*
More specific subject area:	Plant grafting
Name of your method:	Cassava grafting using top wedge technique and healing grafts in a wooden chamber
Name and reference of original method:	De Bruijn, G. H., Dharmaputra, T. S., 1974. The Mukibat system, a high-yielding method of cassava production in Indonesia. *Netherlands Journal of Agricultural Science*, 22(2), 89–100. https://doi.org/10.18174/njas.v22i2.17226. Souza, L. S., Diniz, R. P., de Jesus Neves, R., Alves, A. A. C., de Oliveira, E. J., 2018. Grafting as a strategy to increase flowering of cassava. *Scientia horticulturae*, 240, 544–551. https://doi.org/10.1016/j.scienta.2018.06.070.
Resource availability:	Not applicable).

## Background

Cassava (*Manihot esculenta* Crantz) is a perennial plant belonging to the Euphorbiaceae family [1] and native to Latin and South America [2]. It is widely grown in the tropical and sub-tropical regions of Africa, Asia, and Latin America for its starchy root tubers. According to FAOSTAT (2022), about 314.8 million tons of cassava are produced globally and Nigeria, the world's highest cassava producer contributed about 63.0 million tons representing 19.82 % of global production [3]. It plays an important role as a staple food in the diet of over 500 million people in developing countries particularly in Africa [4]. Among all root crops, cassava is widely distributed and cultivated in most parts of Africa such as Ghana [4]. Thus, it is considered one of the world's predominant carbohydrate sources for human diet [5].

For example, in Ghana, cassava is one of the most important crops and over 70 % of farmers are engaged in its production [6]. According to FAOSTAT (2022), Ghana contributed over 21.8 million tons representing 7.21 % and 11.3 % of global and Africa's cassava production respectively [3]. This eventually contributed to about 22 % and 30 % of the agricultural Gross Domestic Product (AGDP) and the daily calorie intake of most Ghanaians, respectively [7]. The cassava tuber flesh, peels and leaves are also used as livestock feed [8]. Moreover, the starchy tuberous cassava roots are used in making essential industrial products such as sweeteners, beverages, cosmetics, medicine, biodegradable materials, bioethanol, textiles and plywood, thereby becoming one of the fastest growing industries worldwide. Also, processed cassava is used as raw materials for producing non-traditional products such as starch, flour, chips and pellets which enhances income generation and improves the livelihood of farmers and poor urban inhabitants [1].

The increase in demand for cassava for local, industrial and export purposes call for an urgent need to develop or introduce high throughput techniques such as molecular marker assisted selection (MAS), genome sequencing and genome editing, tissue culture micropropagation and remote sensing to complement the improvement of cassava production. However, these techniques have their own challenges including high cost of implementation, complex adoption methods and limited relevance to small scale farmers [9]. Also, multiplication rate is slow and this prolongs the breeding cycles in cassava, therefore, a minimum of eight years is estimated to develop a new variety [10]. Grafting has been in use since at least 1000 BCE for agricultural and horticultural purposes, including vegetative propagation and disease resistance. It is the act of joining cambiums of a scion placed on rootstock to form a complete plant [11]. This principle governing the union is based on the capabilities of the meristematic tissues with undifferentiated cells to undergo cell division [12]. Grafting has been widely used in woody plants such as *Hevea brasiliensis* [13], *Carya illinoensis* [14], Citrus spp. [15,16], *Vitis vinifera* [17] and can be applicable to enhance cassava propagation. For instance, in fruit tree production, grafting techniques have been applied to improve resistance to diseases, maintain desirable traits, improve tolerance to stress, enhance pollination and improve fruit quality [18]. Furthermore, grafting has been used to improve production of vegetables, particularly in the *Cucurbitaceae* and *Solanaceae* families. It has been successfully adopted and used to improve resistance to biotic (Nematode, Fusarium wilt) and abiotic (drought and salinity) stresses, increase yields and fruit quality [[19], [20], [21]].

In *Euphorbiaceae* family, grafting was first performed by Mukibat, an Indonesian farmer living on the island of Java [22]. This improvised grafting technique termed as ‘Mukibat Technique’ was facilitated by the insertion of a thin piece bamboo into the pith of scion and rootstock to enhance successful grafting. The technique significantly enhanced yield from 30 to 100 % compared to intact cassava plants [22]. Since then, grafting techniques such as chip budding, splice and cleft grafting has been used for several purposes such as to improve growth and root yield in Asia (Thailand) [23,24], and induce earlier branching and flowering in cassava in South America (Brazil) [25,26]. Cassava is susceptible to a suite of plant pathogens including viruses, bacteria, and fungi as well as insect pests, leading to low production levels and food insecurity threats [27]. Furthermore, abiotic constraints such as drought, nutrient deficiencies, and soil degradation remain the main reasons for low cassava production [28]. Therefore, grafting can be used to introduce resistant traits from wild cassava relatives and/or resistant cultivars to address the crop's susceptibility to various biotic and abiotic stresses. Also, the combination of desirable traits of different cassava varieties, including early and high flowering rates, drought tolerance, nutrient use efficiency, and adaptability to marginal growing conditions can be employed through grafting [25]. Grafting has been established as a valuable scientific research tool to study the long-distance transport of signaling molecules in plants [29,30]. In cassava, grafting can facilitate the study of genetic interactions and genetic material transfer between different cassava genotypes, contributing to our understanding of the crop's genetic diversity and possible improvement. Thus, it is evident that the demand for cassava grafting is driven by both practical production challenges and pertinent scientific research imperatives.

However, the prospects of grafting have not been fully explored in cassava cultivation, especially in Africa. Thus, extensive research on cassava grafting is scarcely available. This could be due to the unavailability of a detailed cassava grafting protocol hindering its widespread adoption and further exploration for the advancement of cassava cultivation and research. Availability of a detailed and standardized cassava grafting protocol will complement breeding programs. This will ensure the sustainable utilization of cassava germplasm by enhancing growers’ ability to develop their own cassava cultivars with desirable traits including erect, early flowering and high yielding [31]. Some limitations recognized in previous cassava grafting studies includes the use of plastic covers for graft healing [25] which may result in poor relative humidity regulation and compromise graft success rate [32]. High and constant relative humidity ensures decreased transpiration rate and dying of scions [32]. The current study employs a wooden healing chamber as an alternative to plastic covers which do not only provide a controlled environment for graft healing but also mitigates external stressors. It provides a suitable environment for extended graft healing period required by tree crops such as cassava. Moreover, previous reports on cassava grafting lack a detailed overview and adequate illustrative aids for the process, making it difficult to replicate and hindering the overall advancement of the technique. This study presents a step-by-step cassava grafting protocol with illustrative aids. The use of cassava seedlings raised from cuttings in this study offers a more manageable plant size which facilitates precision during the grafting procedure and ensures uniformity compared to the use of bigger plantlets [24]. In view of this, the study attempts to establish a robust cassava grafting protocol, facilitating the large-scale propagation of grafted cassava cultivars for cultivation through the implementation of an optimized top wedge grafting technique and a wooden healing chamber.

## Method details

### Reagents

• 70 % ethanol (See Recipes)
• Bleach solution (See Recipes) (Powerzone®, CEVAG, Tema, Ghana)
• Distilled water.

### Supplies and tools

• Razor blades (Euromax Platinum, Japan)
• Disposable gloves (RONCO, Ontario, Canada)
• Mist bottle (Myers Associates, Washington Ave, USA)
• Homemade wooden healing chamber
• Grafting clips (Johnny's Selected Seeds, Winslow, Maine, USA)
• Soilless media: potting mix (Fertiplus Universal Potting Soil, Berlin, Germany)
• Nursery bags (10 cm × 25 cm) (Agriseed Ltd, Adabraka, Accra, Ghana)
• Fungicide (Agrithane® Mancozeb 80WP, AGRIMAT Ltd, Acca, Ghana)

### Equipment

• Data logger (Temtop TemLog 20H [Elitech Technology, Silicon Valley, CA])
• Digital Vernier caliper (Neiko 01407A Stainless Steel Electronic Digital Caliper; Ridgerock Tools Inc., USA).

### Constructing a homemade wooden healing chamber

#### Supplies and tools

• 1-inch wooden boards
• Nails
• Black and white plastic sheets
• Flexible metal rods

1. Construct a frame with dimension 1.4 (L) × 1.0 (W) × 0.4 (H) m using the 1-inch wooden board. The inside of the wooden frame (bottom floor) should be lined with a black plastic sheet as illustrated in Fig. 1a to keep the wood from being soaked with water and to also block the incidence of solar radiation.

**Fig. 1:**
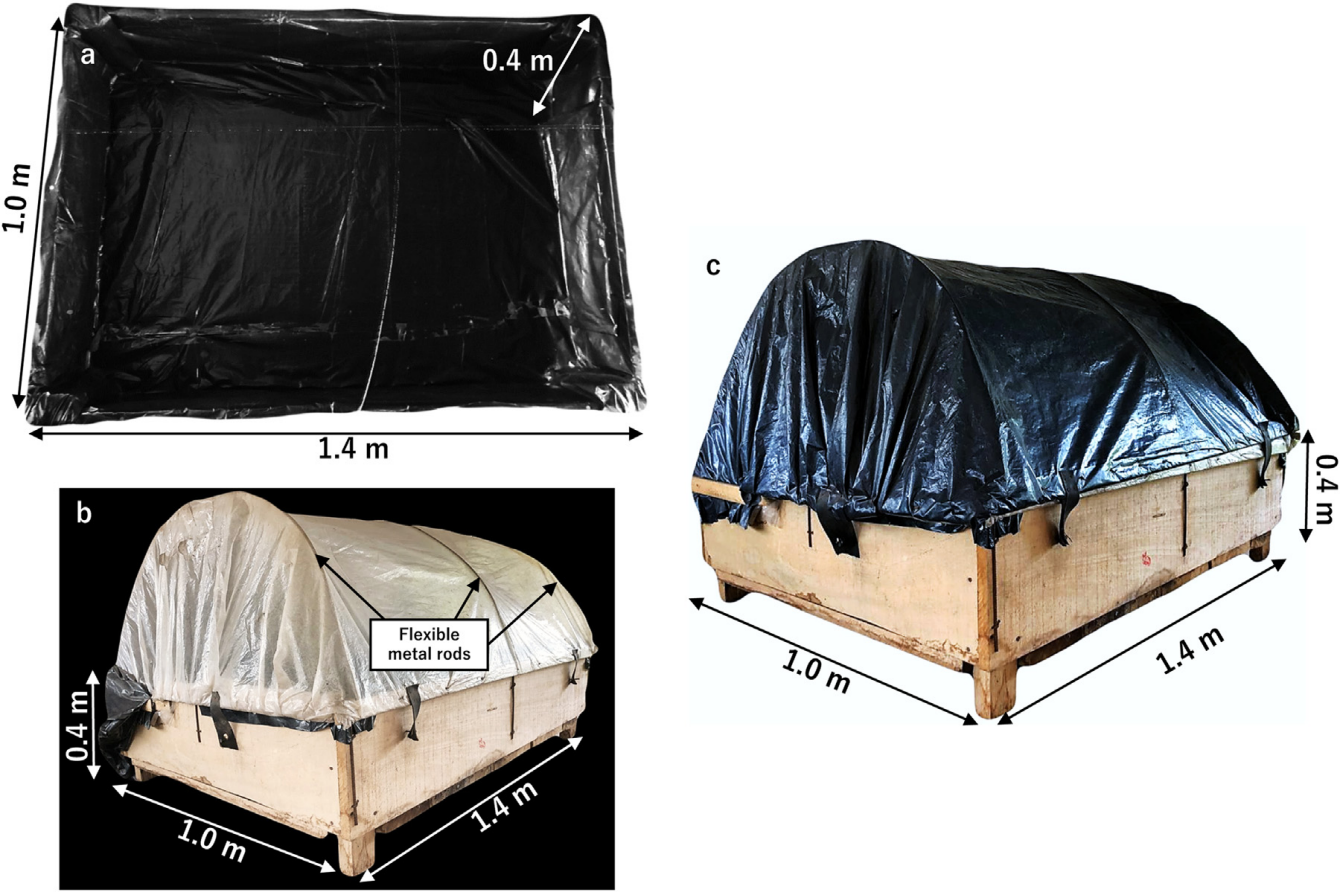
Constructing a homemade wooden healing chamber. (a) A 1.4 (L) × 1.0 (W) × 0.4 (H) m frame constructed using a 1-inch wooden board and bottom floor lined with a black plastic sheet. (b) Flexible metal rods fixed on the frame and covered with a layer of white/transparent plastic sheet. (c) Frame covered with an additional layer of black plastic sheet to create shade.
2. Fix three flexible metal rods on the chamber and cover the structure with a layer of white/transparent plastic sheet (Fig. 1b). Afterwards, a layer of black plastic sheet (Fig. 1c) is to be used to cover the frame to block available sunlight from entering the chamber.
3. Ensure constant humidity of 80–95 % and temperature of 25–35 °C inside the chamber before grafting.

### Establishment of cassava nursery

1. Obtain cassava hardwood stem cuttings of length 10–15 cm from 9 to 12 months-old, matured cassava plants with sturdy stems and well-formed leaves (Fig. 2a, b). These plants are raised by means of matured stem cuttings propagation.

**Fig. 2:**
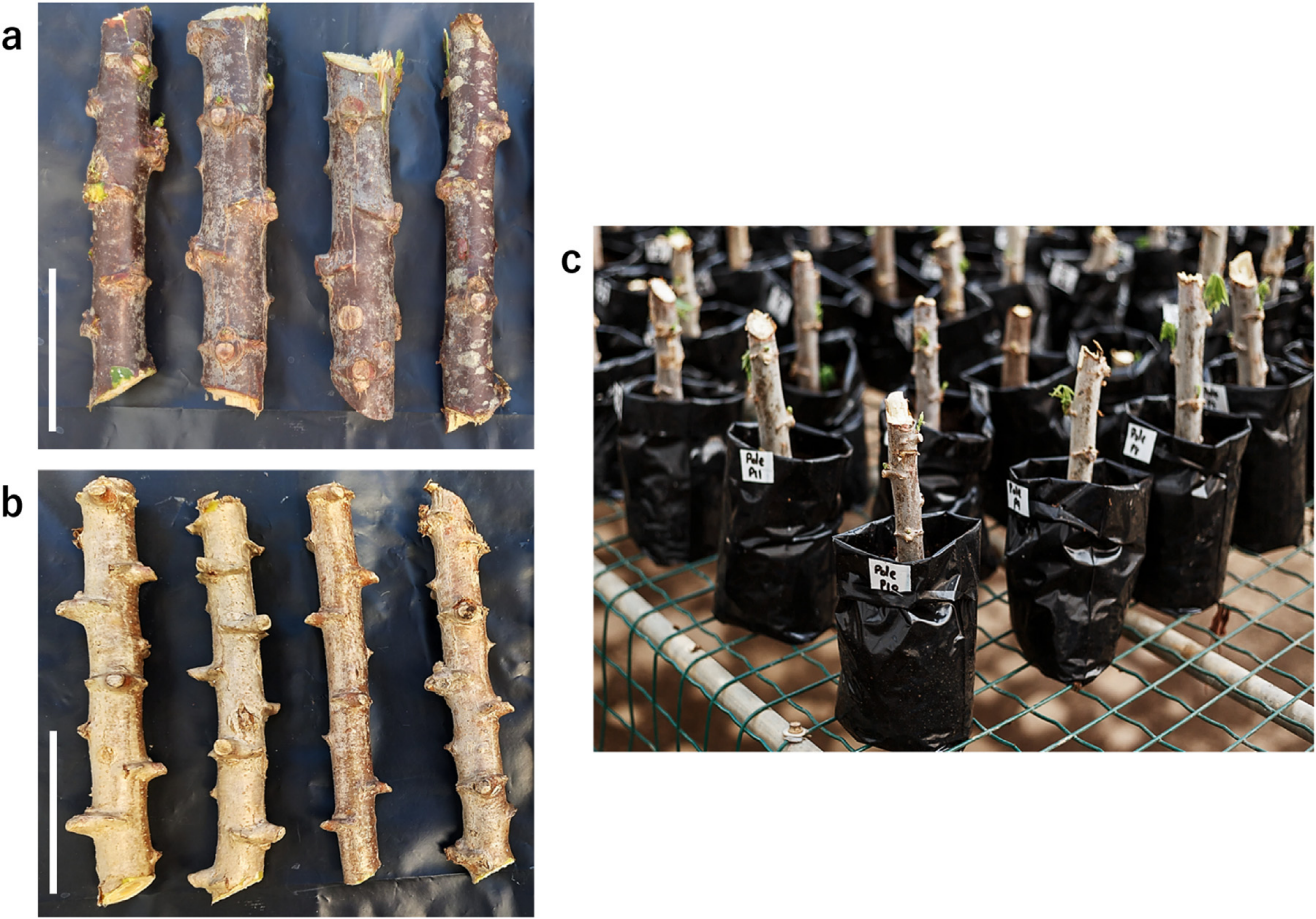
Nursery establishment using stem cuttings of length 10–15 cm obtained from matured cassava cultivars. (a) Representative cuttings from “Pole Bankye” used as scion. (b) Representative cuttings from “CRI-Bankye Hemaa” used as rootstock. (c) Raising healthy cassava seedlings under greenhouse conditions. Bars: 5 cm.
2. Prepare a fungicide solution by mixing 75 g of Mancozeb® in 15 L of water and dip the cassava cuttings into the solution before planting in the nursery medium.
3. Fill perforated nursery bags of dimensions (3.94“ × 9.84”) with potting mix. Plant the disinfected cuttings and water immediately.
4. Raise healthy cassava seedlings under controlled and optimum conditions with about 50 % shade, 20 to 35 °C temperature and 50 to 80 % humidity (Fig. 2c).
5. After approximately 6 weeks of establishment, select scions and rootstocks with similar girths for grafting.

The procedures for steps 1–2 under establishment of cassava nursery are shown in Video 1

The procedure for step 3 under establishment of cassava nursery are shown in Video 2

The procedures for steps 4 under establishment of cassava nursery are shown in Video 3

### Procedure for cassava grafting

1. **Sterilization of grafting tools and equipment**

Sterilize razor blades and grafting clips with 70 % ethanol and 10 % bleach to avoid pathogen contamination. Also, sanitize hands with 70 % ethanol.

2. **Grafting of cassava seedlings**

**Fig. 3:**
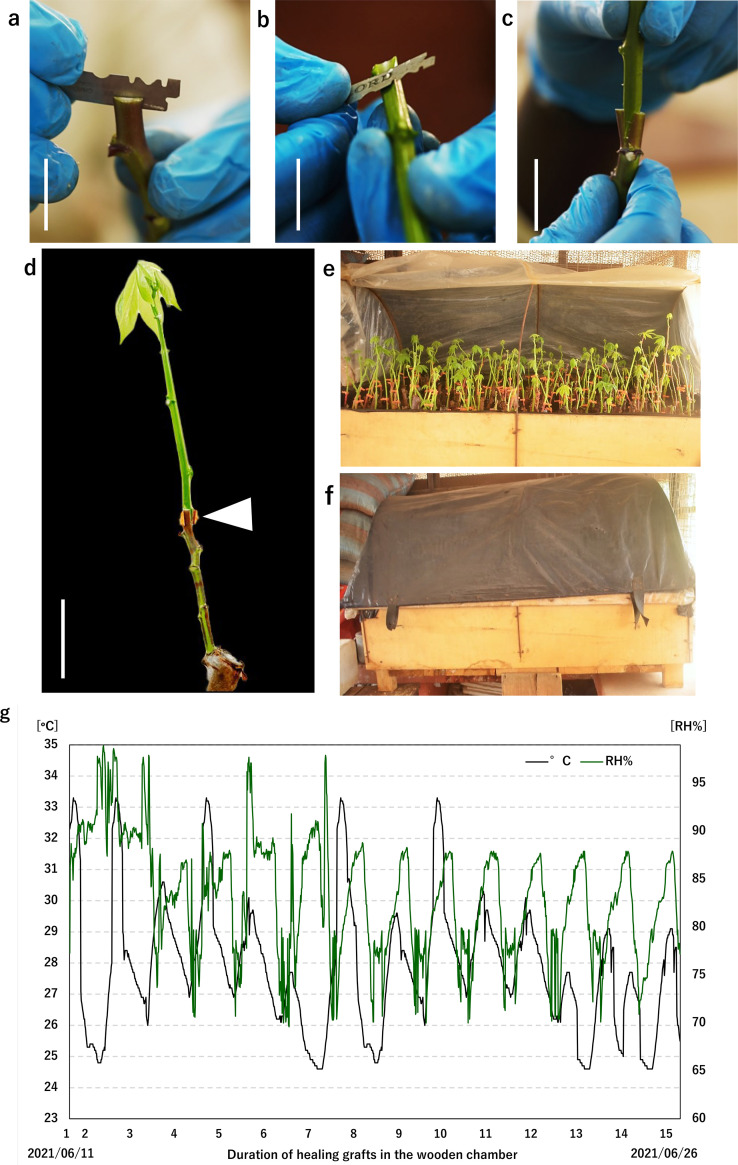
Prepared and assembled scion and stock plants. (a) A cassava stock “CRI-Bankye Hemaa” prepared by creating a 1 cm vertical slit. (b) Cassava scion “Pole Bankye” prepared by cutting the stem into a wedge shape. (c) Scion firmly inserted into the vertically slit stock plant. (d) Stock and the scion plant with small young leaves at the apical portion assembled by firmly securing grafting joint with a grafting clip. (e,f) Grafted seedlings placed into the homemade wooden healing chamber and covered to heal. (g) Healing chamber conditions logging summary. Relative humidity (%) and Temperature (°C) in the wooden healing chamber, during graft healing period. Data points: 1323; Elapsed time: 15 days; Highest: 33.3 °C/98.9 %; Lowest: 24.6 °C /69.6 %; Average: 27.9 °C/83.1 %. Bars: 5 cm.
d. Cut the scion into a wedge shape and carefully insert it into the vertically slit rootstock while ensuring maximum cambial region alignment by firmly securing the grafting joint with a grafting clip (Fig. 3b–d).
e. Prune all the leaves on the rootstock and maintain only two small young leaves at the apical portion of the scion. This will help reduce the rate of transpiration (Fig. 3d).
f. Place grafted seedlings into the homemade wooden healing chamber and maintain high humidity by misting the inside immediately with clean water (Fig. 3e).
g. The procedures for steps a-f under section 2 are shown in Video 4

Prior to grafting, the vegetative growth rate of the cassava seedlings was evaluated. Stem diameter, plant height and number of leaves were measured at 42 days after planting cuttings (DAP).

a. Select 5–week–old cassava seedlings with a stem diameter of approximately 6–10 mm, a stem height of 20–30 cm and at least 10 leaves. The thicker stems prevent stem breakages, make the grafting process easy and increase the graft success rate.
b. Irrigate the cassava seedlings adequately before grafting.
c. Use the cleft or top wedge grafting method by creating a 1.0 cm vertical slit in the rootstock (Fig. 3a).

3. **Healing of grafted cassava seedlings in a homemade wooden healing chamber**

a. The provision and regulation of optimum humidity, temperature and light are essential for the reconnection of vascular tissues between grafted scions and rootstocks. This ensures an adequate supply of nutrients and moisture to the scion and this process essentially occurs in a healing chamber.
b. Identify a proper placement of healing chamber in a warm storage environment such as indoor, greenhouse and lath houses.
c. Mist the chamber with 150 mL of clean water and cover the chamber first with a white plastic sheet before the black plastic sheet to create a controlled environment for the grafted plants to heal (Fig. 3f). The misting helps maintain a high humidity level (80 to 95%) and prevent plants from drying out. The white plastic sheet helps to reflect light and prevent direct sunlight from reaching the plants, while the black sheet helps to absorb light to ensure a 25–35 °C temperature inside the chamber, creating a warm and stable environment for the plants to heal (Fig. 3g). This helps to promote a successful graft healing process and reduces the risk of failure.
d. Leave the chamber completely closed for the first 2–3 days. Subsequently open and mist the chamber frequently for approximately 12–13 days. Carefully inspect the grafted plants anytime you open the chamber to ensure that the scions and rootstocks are still intact. Remove any adventitious root in the scion's using scissors.
e. Avoid misting grafts directly especially at the graft junction whiles they are in the chamber to prevent excess water on the grafts which results in the removal of the scion. Instead, water plants by placing plants in clean water for 2–5 min to ensure that the water is drawn upward into the potting soil.
f. After 15 days after grafting, the grafted plants should be moved to a greenhouse for acclimatization by gradually introducing them to light and low humidity levels for 30 days (Fig. 5a). At this stage, watering or irrigation must be done carefully to prevent damage to the graft union (junction).
g. Remove the grafting clips after 30 to 40 days when the union is strong and graft take is successful (Fig. 5a and b).
h. Transplant grafted plants to the field 50–60 days after grafting using a planting distance of 1.0 m (inter-row) x 0.9 m (intra-row) and all necessary and recommended crop management and cultural practices must be carried out.

The procedures for steps a-b under section 3 are shown in Video 5

### Data collection and analysis to evaluate the viability of the grafting protocol

Prior to grafting, the vegetative growth rate of the cassava seedlings was evaluated. Stem diameter, plant height, number of leaves and chlorophyll content (Apogee Instruments Inc. Logan, UT, USA) were measured at 42 days after planting (DAP) cuttings. The data were then subjected to independent t tests (α=0.05) analysis and results presented as mean ± standard error. Statistical analyses were performed in Genstat V12 statistical package (VSN International (2022). The different scions and rootstocks combinations gave rise to 4 treatments: (1) “Pole Bankye”/ “Pole Bankye” homograft; (2) “CRI-Bankye Hemaa”/ “CRI-Bankye Hemaa” homograft; (3) “Pole Bankye”/ “CRI-Bankye Hemaa” heterograft and (4) “CRI-Bankye Hemaa”/ “Pole Bankye” heterograft. At 60 days after grafting, the survival rate (graft success index) was evaluated in three independent tests considering vigorous grafted cassava seedlings with increased shoot length and development of new leaves. The data were transformed to the percentage of survival (%).

## Method validation

**Fig. 4:**
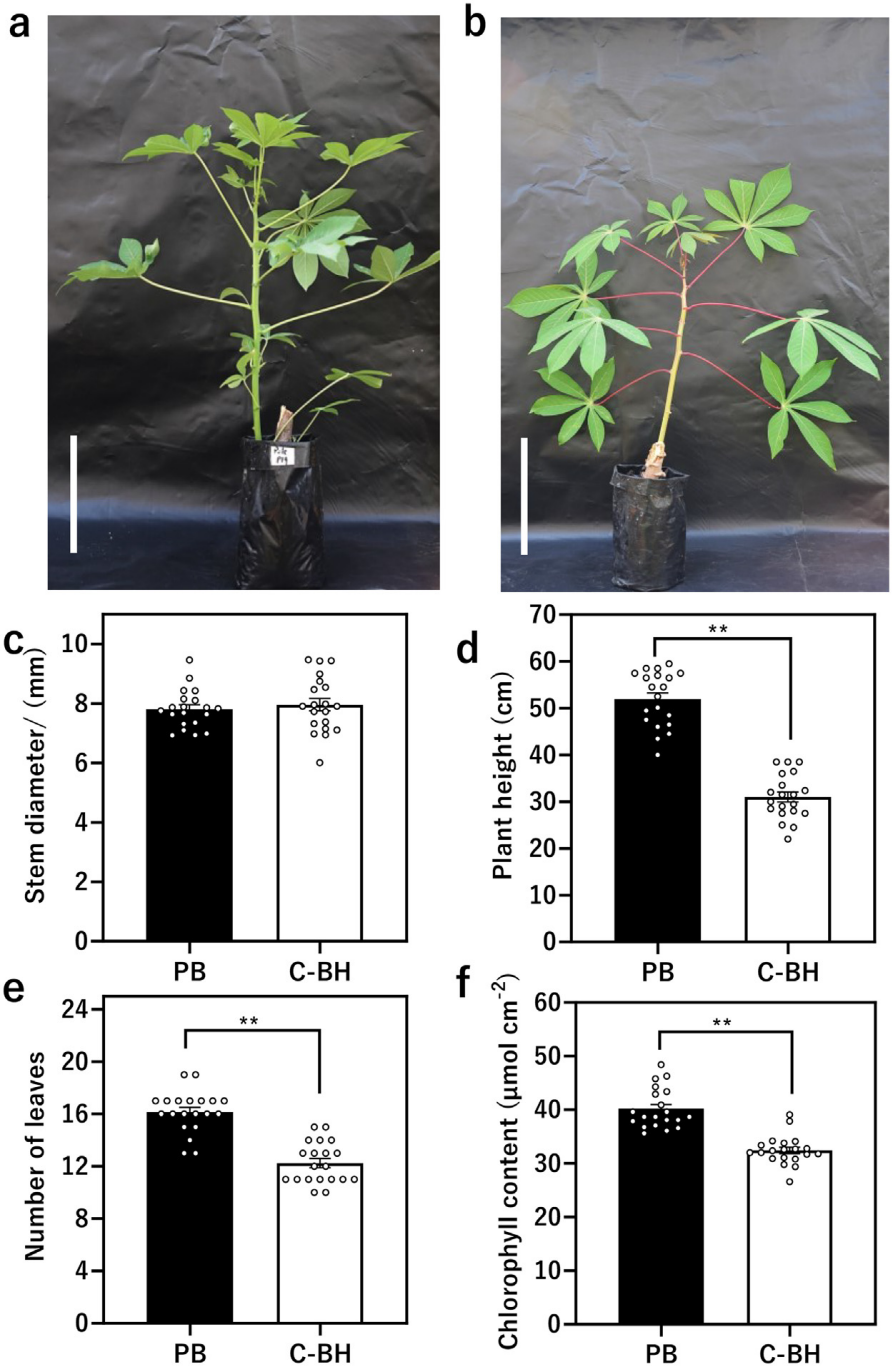
Evaluation of the vegetative growth rate of cassava seedlings prior to grafting. (a-b) Photographs of stock and scion plants; “Pole Bankye” and CRI-Bankye Hemaa at 42 DAG. Scale bars, 5 cm (c-f) Measurement of stem diameter, height, number of leaves and chlorophyll content of cassava seedlings at 42 DAG. PB, Pole Bankye; C—HB, CRI-Bankye Hemaa. Error bars indicate the means ± SE. Asterisks represents significant differences determined by Student's T-test (*P < 0.05; ** *P* < 0.01).

Grafting, the act of explanting a scion onto a rootstock has been a useful technique in the fields of agricultural and plant science. While grafting has been widely adopted in various horticultural crops, its application in cassava has been limited, notably, in Africa. In response to this gap, we present the first report of an optimized cleft grafting protocol for cassava developed in Africa. The development of an optimized cassava grafting protocol with illustrative elements including stepwise photos and videos that allows reliable grafting is essential to obtaining reproducible results with high success rates. To observe the optimum growth and vigor status of the cassava seedlings for grafting, the vegetative growth of the cassava seedlings at 42 DAP was measured. The cassava seedlings (Fig. 4a–b) exhibited growth in stem diameter (6 mm – 9 mm), plant height (30 cm – 50 cm), number of leaves (12 – 19) and chlorophyll content (26.6 µmol cm-2 – 48.4 µmol cm-2) (Fig. 4c–f). These agronomic parameters were suitable for grafting success.

**Fig. 5:**
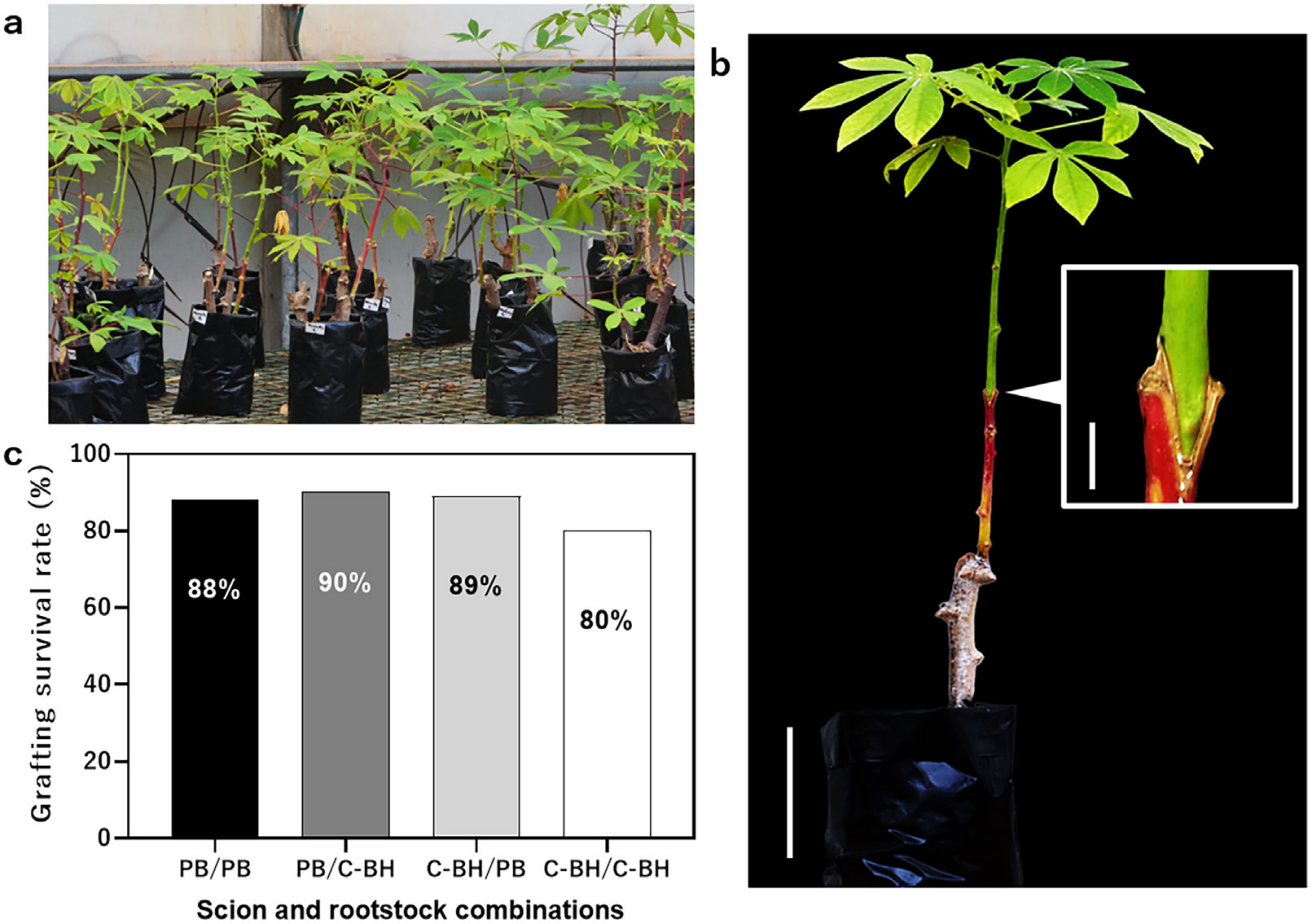
Post grafting acclimatization and evaluation of 30 days old cassava grafts under greenhouse conditions. (a) Grafted plants acclimatized under greenhouse conditions without grafting clips at the graft junctions. (b) Representative “Pole Bankye”/”CRI-Bankye Hemaa” successful graft at 30 DAG. The “Pole Bankye” cassava scion developed new leaves. Arrowhead indicates the graft junction. (c) The survival rates were determined 30 days after grafting and acclimatization of 19–26 grafts for each combination under greenhouse conditions based on grafts with apparent graft-take, new leaves and good vigor. PB, Pole Bankye; C-BH, CRI-Bankye Hemaa. Bars: 1 and 5 cm.

Following these optimized conditions, a total of 4 reciprocal graft combinations using two cassava cultivars were prepared as follows for their graft survival rate evaluation: (1) “Pole Bankye”/“Pole Bankye” homograft; (2) “CRI-Bankye Hemaa”/“CRI-Bankye Hemaa” homograft; (3) “Pole Bankye”/“CRI-Bankye Hemaa” heterograft and (4) “CRI-Bankye Hemaa”/“Pole Bankye” heterograft. The success rates were 88 % (*n* = 25), 80 % (*n* = 26), 90 % (*n* = 20) and 89 % (*n* = 19), respectively (Fig. 5c).

In this study, grafting conditions were optimized by preparing a homemade wooden healing chamber (Fig. 1) and controlling specific environmental conditions such as temperature, humidity, light, and water for grafted cassava (*M. esculenta*) seedlings, viz, temperature (25–35 °C), humidity (70– 98%) (Fig. 3g), no direct light and plants kept hydrated through regular watering or misting. In the view of practical agricultural use, we further focused on the “Pole Bankye”/“CRI-Bankye Hemaa” heterograft combination. In the second grafting experiment for this heterograft combination, the grafting survival rate was 90 % (*n* = 29). Thus, the high success rates were achieved in reciprocal graft combinations of two cassava cultivars, indicating the applicability of the optimized grafting protocol. This current study therefore provides an easy to follow and robust protocol for routine cassava grafting with high success rate. In future practices, grafting success rate could be affected by the anatomical and physiological differences in the genotypes related to their stem diameter as well as genetic incompatibility during the grafting process. The cleft grafting technique is an effective method for the preparation of different cassava scion and rootstock combinations in the view of anatomical differences in the genotypes. In contrast to other published grafting procedures for cassava, Souza et al., [25] reported a total average grafting survival rate of approximately 70%, viz, 60 % to 82 % whiles detailed grafting procedure and survival rates were not adequately provided in the report by Bangthong et al., [24]. However, our optimized graft healing conditions using the wooden chamber significantly improved graft success rates of cassava cultivars, achieving survival rates of up to 90%. The cleft grafting method was used in the current study and this choice of grafting method was based on a previous test in tree crop species such as *Plukenetia volubilis, Tamarindus indica, Mangifera indica* and in *Manihot esculenta* where the cleft grafting method resulted in higher survival rate compared to the other two methods of grafting including splice grafting and budding [25,[33], [34], [35]]. In contrast to the study by Souza et al., [25] where grafts were bagged with a transparent plastic bag after the grafting procedure, in the current study, grafted cassava seedlings were healed in a homemade wooden healing chamber. Wood is a hygroscopic material which possesses hygrothermal properties with the ability to respond to changes in humidity and temperature [36]. Studies have shown that maintaining the optimum temperature and humidity is crucial for grafted plants to successfully heal such that high humidity prevents transpiration of the grafted plants [37]. The properties of this natural insulator help to maintain the temperature and humidity inside the healing chamber ensuring that the grafted plants are protected from fluctuation in conditions which can cause the graft to fail. Additionally, the use of a wooden healing chamber coupled with the regularized opening and closing of the chamber provides a more balanced and ventilated healing environment for grafted plants and may minimize the risk of fungal infections. Moreover, the scalability and adaptability of the wooden chamber based on the specific needs of the grafting operation makes it even more flexibly advantageous to use compared to the use of plastic bag covers. These stable conditions ensure a more favorable and consistent healing process for the graft union. This resulted in an improved survival rate of grafted cassava seedlings which were successfully transferred to field conditions. We evaluated successful grafting by examining post-grafting traits including scion plants staying alive at 6 weeks after grafting and continuous growth of scion. Compared to previous studies, our grafting success was observed over an extended time period until vigorously grafted plants were successfully transplanted into the soil. This is because graft success rates are lower at a later time point than after a few days [38]. Also, delayed grafting compatibility may be observed during graft healing process and may be dependent on the combination of cultivars, plant growth regulators, light conditions and photoperiod manipulations [26].

Cassava is a tree crop species that demands an extended healing period to ensure successful graft union formation as compared to herbaceous plants. This is attributed to the woody nature of cassava, which necessitates more time for the vascular tissues to integrate and for the plant to establish a robust connection between the rootstock and the scion [39]. The advantages of this longer healing time include the development of a strong and durable graft union, which is essential for the long-term growth and productivity of the grafted cassava plants. Additionally, the extended healing period allows for the proper establishment of the vascular network, ensuring efficient nutrient and water transport throughout the plant [40]. The use of a wooden healing chamber provides a stable and protective environment for the grafted seedlings without the use of sophisticated equipment's such as humidifiers [41]. This stability is crucial for the gradual integration of the scion and rootstock, promoting a more secure and lasting graft union. The consistent protection offered by the chamber shields the graft from external stressors and disturbances. Our grafting protocol is practical and applicable for high success rate which could be a useful tool to compliment cassava improvement programs in Africa. Another advantage is its suitability for molecular studies on long-distance transport of signaling molecules such as small RNA, mRNA and proteins between different cassava cultivars. It is worth noting that fine-tuning of cassava grafting technique could be beneficial in managing biotic and abiotic stresses as well as improving tuber yield. Improving cassava yields and resilience is crucial for food security in Africa, where it is a staple crop. Our grafting protocol can contribute to these efforts by enabling new research directions on systemic signaling, stress responses, and yield improvement. Further applications of the cassava grafting protocol developed among different genotypes is essential for its adoption, sustainable cassava cultivation and optimization of agricultural outcomes. Also, further investigation of other grafting methods, such as chip-grafting and splice grafting, will further enhance the efficiency of the grafting procedure.

This study presents the development of a cassava grafting protocol in Ghana, Africa. In this study we present a simple and efficient grafting protocol for cassava, demonstrating a step-by-step procedure with illustrative aids and optimum conditions to generate a high grafting success rate of approximately 90%. Specifically, graft healing conditions have been optimized by developing a wooden chamber for the healing of grafted cassava plants. This improved the graft survival rate as compared to results in previous studies that used plastic bags. The optimal temperature and humidity conditions for graft survival reported in this study can serve as a benchmark for future cassava grafting efforts. The protocol developed using a top wedge grafting technique for cassava (*M. esculenta*) cultivars is practically applicable in fields conditions with high reproducibility and success rates. The availability of such an efficient protocol will be a versatile tool to benefit cassava researchers to fully explore research prospects and significantly contribute to breeding efforts. Cassava farmers can also develop their unique cassava combinations that suit their specific preferences, environmental conditions, and market demands.

### Limitations

1. The selection of scions and rootstocks with similar girths is necessary to ensure maximum cambium alignment and to achieve high survival rates.
2. Good sterilization of cutting equipment, grafting clips and working surfaces is necessary to prevent bacterial or fungal contaminations during graft recovery. Washing of hands and wearing hand gloves before carrying out the grafting procedure is highly recommended.
3. The choice of grafting method is very critical in cassava grafting and the cleft grafting presented a higher survival rate is the optimum method to use compared to the splice.
4. Another distinguishing feature of this protocol is the removal of all the leaves on the rootstock and maintaining two small young leaves at the apical portion of the scion. This will help reduce the transpiration rates of the graft until establishment of graft union and development of new leaves. This also ensures that long leaf petioles do not disturb grafts in the healing chamber since the graft junction is fairly unstable at the initial stages of the healing period though they are being held with grafting clips.
5. The healing chamber must be frequently opened and misted to ensure adequate aeration, which is important for the long-term healing of grafts. In our case, we opened the chamber at 4 h interval during the day to facilitate air circulation, allow for the regulation of humidity and temperature within the chamber and also prevent the buildup of excess moisture that could lead to fungal growth.
6. Ensure that no water is sprayed on the graft union parts before joining the two together and avoid overhead watering of plants to minimize the formation of adventitious roots as well as to prevent water soak at the graft junction which can result in unsuccessful graft-take. Similar observations were made in *A. thaliana* seedling grafting experiments reported by Notaguchi et al., [42] and Marsch-Martinez et al., [43].

## Ethics statements

Not applicable.

## CRediT authorship contribution statement

**Frank Opoku-Agyemang:** Conceptualization, Methodology, Formal analysis, Investigation, Writing – original draft. **Jacqueline Naalamle Amissah:** Conceptualization, Methodology, Investigation, Resources, Writing – original draft, Writing – review & editing. **Stella Owusu-Nketia:** Investigation, Resources, Writing – review & editing. **Peter Amoako Ofori:** Conceptualization, Methodology, Formal analysis, Investigation, Resources, Writing – original draft, Writing – review & editing, Supervision, Funding acquisition. **Michitaka Notaguchi:** Conceptualization, Methodology, Investigation, Resources, Writing – review & editing, Supervision, Funding acquisition.

## Declaration of competing interest

The authors declare that they have no known competing financial interests or personal relationships that could have appeared to influence the work reported in this paper.

## Data Availability

Data will be made available on request.

## Appendix Supplementary materials

Image, video 1

Image, video 2

Image, video 3

Image, video 4

Image, video 5
